# A Study of Lock-Free Based Concurrent Garbage Collectors for Multicore Platform

**DOI:** 10.1155/2014/237356

**Published:** 2014-06-30

**Authors:** Hao Wu, Zhen-Zhou Ji

**Affiliations:** School of Computer Science and Technology, Harbin Institute of Technology, Harbin 150001, China

## Abstract

Concurrent garbage collectors (CGC) have recently obtained extensive concern on multicore platform. Excellent designed CGC can improve the efficiency of runtime systems by exploring the full potential processing resources of multicore computers. Two major performance critical components for designing CGC are studied in this paper, stack scanning and heap compaction. Since the lock-based algorithms do not scale well, we present a lock-free solution for constructing a highly concurrent garbage collector. We adopt CAS/MCAS synchronization primitives to guarantee that the programs will never be blocked by the collector thread while the garbage collection process is ongoing. The evaluation results of this study demonstrate that our approach achieves competitive performance.

## 1. Introduction

Garbage collection mechanism has been widely used in modern object oriented programming languages such as Java or C#. The garbage collectors can guarantee the security and reliability of the runtime systems, but they also introduce additional performance overhead. Traditional garbage collectors perform the entire memory reclamation by suspending the running programs [1]. It is obvious that such a strategy will seriously affect performance and responsiveness of the systems, and this is especially unacceptable for a real-time system. The emergence of multicore architectures has a profound impact on the implementation of programming languages, and the languages that support garbage collection mechanism should make use of this parallel processing capacity [2]. With the development of multicore architectures, the designing of parallel and concurrent garbage collector faces a number of chances and challenges.

Kliot et al. [3] thought that the most critical performance issue for modern on-the-fly collectors is the stack scanning phase. Most of the garbage collectors will interrupt the mutator threads when performing the scanning of the runtime stack in order to obtain an accurate heap snapshot which will be used in the subsequent marking phase. Traditional blocking stack scanning strategy may lead to a long and unpredictable pause time.

Another noteworthy performance issue is heap fragmentation. As we know, most of modern on-the-fly garbage collectors adopt the strategy that does not move the objects in order to obtain a less pause time during the collection process. One of the major drawbacks of that strategy is that the heap becomes fragmented and the allocation becomes more costly for long time running programs. To solve fragmentation problem, an extra heap compaction function is added into the garbage collection. Apparently, this compaction function should be carefully designed to avoid creating more overhead.

In this paper we mainly study two issues of designing concurrent garbage collectors, stack scanning and heap compaction. In the multicore computing environments, lock-based synchronization mechanism does not scale well with concurrent execution threads. To solve this problem, lock-free algorithms are designed for highly concurrent systems. Compare and swap (CAS) primitive can be used to execute atomic read-modify-write operations on shared data in lock-free algorithms. Multiword compare and swap (MCAS) is an extension form of CAS which supports CAS operations on a number of memory locations simultaneously. We first use CAS to design a concurrent stack scanning mechanism. Then for heap compaction, we use MCAS synchronization mechanism to design a lock-free and concurrent object copying process.

The rest of this paper is organized as follows. In Section 2 we describe a methodology of concurrent stack scanning. In Section 3 we present a design of concurrent heap compaction. Measurements are reported in Section 4 and related work is discussed in Section 5, and finally we conclude in Section 6.

## 2. Concurrent Stack Scanning

In this section we present a concurrent stack scanning mechanism using CAS synchronization primitive, which allows collector threads to scan the stack concurrently with mutator threads in lock-free manner. CAS is supported in hardware by most processor architectures. The abstract definition of CAS is showed in Algorithm 1 and “atomically” indicates that the function is to be executed instantaneously. This primitive takes three arguments: a memory location, an expected old value, and a new value to write.

Mark-sweep garbage collection mechanism remains widely used today although its basic algorithm is simple. The collector starts from a set of roots then traverses the graph of objects and marks each encountered object. Then it performs a sweep of the heap and reclaims all unmarked objects. Roots are the pointers from the initial set that will be scanned to find live objects. The scanning procedure must provide an atomic snapshot of the stack for the on-the-fly collectors. Traditional solution of on-the-fly collector works in phases that are separated by handshakes. When entering the stack scanning phase, one of the collectors sends a signal then waits for all the other collectors and mutators to acknowledge it. Then in the following scanning process, mutators are not allowed to access the pointers on the stack. Kliot et al. [3] have pointed that the runtime stack, as the major component of the root set, consumes a large amount of time to be scanned during the garbage collection.

In [4] Auerbach et al. presented an incremental thread stack scanning mechanism by employing a conservative approximation of snapshot. The main objective of the proposed mechanism is to deal with the case of large number of threads. However, for individual thread the scanning process will interrupt the current mutator. Besides that the solution may seriously affect the responsiveness in case a deep stack call is triggered. Therefore, in our design we have put emphasis on the concurrency aspect of the stack scanning.

In order to not to interrupt the mutator thread, the process of obtaining the stack snapshot is divided into the form of sequential scanning of the stack frames. And this scanning process is executed by collector and mutator simultaneously. The scanning results are recorded in a concurrency frame record data structure which we named as* FrameRec*. During whole process of scanning only the mutator thread is allowed to modify the stack, and the collector only collects the information of the encountered pointers then saves the results in the* FrameRec*.

**Figure 1:**
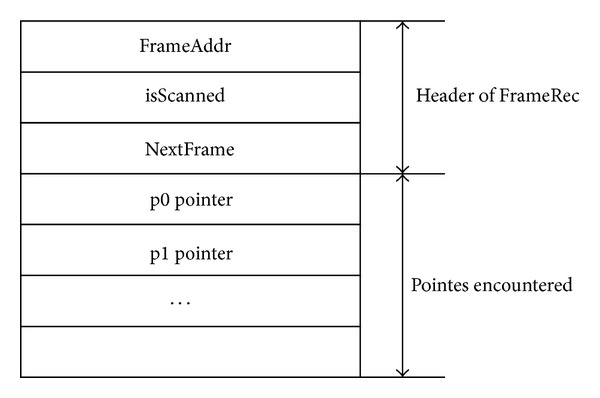
FrameRec structure.

The FrameRec structure is showed in Figure 1, FrameAddr represents the address of the target frame, and isScanned flag indicates whether the corresponding frame has ever been scanned. NextFrame links to a next FrameRec; Pointes region records the pointers that are encountered of the target frame.

When stack scanning process starts, the collector scans the frames in the stack one by one, and then records result in the FrameRec structure. If current visited frame has been scanned, the collector skips and goes on to the next one. When all of the frames are scanned, the scanning process is finished. Then the set of the FrameRec is obtained to be used in the marking phase of garbage collection.

For mutator thread, it will not be interrupted by the collector during the scanning process. However, when entered scanning phase the mutator needs to perform necessary scanning task before leaving the current context of the frame as long as the frame has not been scanned by the collector. Like collector, the mutator saves the scanned results in a newly created FrameRec by the mutator itself. So the time overhead for the mutator is bounded to the scanning of a single frame.

The scanning procedure of collector thread is showed in Algorithm 2. We employ CAS operations to obtain fine-grained synchronization in this process. The first CAS is used to make sure that there is only one header of the FrameRec list when the scanning starts. Failure of the CAS operation indicates that the header has been installed by other collector or mutator thread. In the following scanning, the collector checks the frames one by one. If isScanned flag of the current frame is not set, a CAS operation is employed to set the flag by the collector. After that the collector performs the scanning and records the pointers. Get-Next-Frame procedure searches next frame of the given address and creates a FrameRec structure for the next frame if the frame has no associated FrameRec. Then the next FrameRec to the current one is linked and the scanning process continues until the end of the stack is reached.

## 3. Concurrent Heap Compaction

In this section we present a concurrent heap compaction mechanism using multiword CAS (MCAS) synchronization primitive. The compaction process allows defragmenting of the heap concurrently with the run of mutator thread. Like the previous section, the compaction algorithm is also designed in lock-free manner.

### 3.1. The Challenge

Runtime systems using nonmoving garbage collectors such as mark-sweep will suffer from fragmentation problem after long time running. Because the collectors do not move objects during the collection process, and in this case only increasing the number of collection threads will not alleviate performance bottleneck. And, apparently, a memory defragmenting process is very necessary, which we call heap compaction.

**Figure 2:**
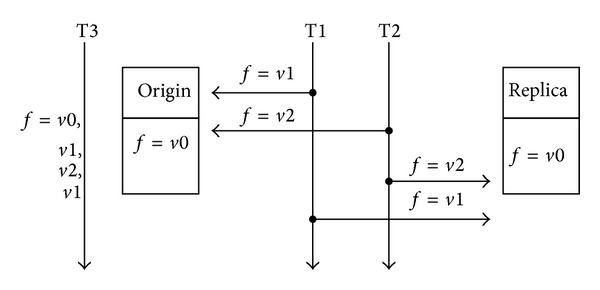
An example of inconsistency scenario during the copying process.

Concurrent compactor allows the mutator thread to modify the object that is being moved by the collector thread. Inconsistency issues must be noticed for there are two copies of an object during the copying process. As showed in Figure 2, origin and replica copies exist simultaneously in the memory until the moving process completes. The thread that accesses the object should maintain the consistency of the two copies. That is the thread must guarantee that the replica contains the same information with the origin when the modification completes. In [1] Pizlo et al. presented an example of inconsistent situation when an object was modified by two threads; here we simply review the process. Figure 2 describes a simple inconsistency scenario that two threads T1, T2 are going to modify the object field *f*. T1 is preempted by T2 which updates *f* with value *v*2 in both origin and replica copies. Actually, the update of T2 is lost, and for a third thread T3 which will get the value sequence *v*0,  *v*1,  *v*2,  *v*1 when it fist accesses origin then with the replica.

Obviously, when there are multiple locations to be updated, the synchronization becomes much more complicated. Since the single word CAS cannot handle multiword synchronization, we consider using multiword CAS to design concurrent object copying. Multiword compare and swap (MCAS) is an extension of normal CAS. It can be used to atomically swap a number of memory locations. The abstract definition of MCAS is showed in Algorithm 3. MCAS operates on N distinct memory addresses, if each address contains the expected old value then all locations are atomically updated with the new values. The function returns true if all of the updates succeed.

MCAS primitive is not directly supported in hardware of modern multiprocessor architectures. We have developed a software-based MCAS which supports arbitrary multiple words synchronization in our previous work. This design can be used for the implementation of nonblocking synchronization for shared variables in multicore or multithread environments. Theoretical analysis and experimental results show that the method could improve the concurrent access performance and provide good scalability. In this work, we adopted it to design the concurrent copying. For details of our MCAS implementation, see [5].

### 3.2. MCAS-Based Extended Object Model

**Figure 3:**
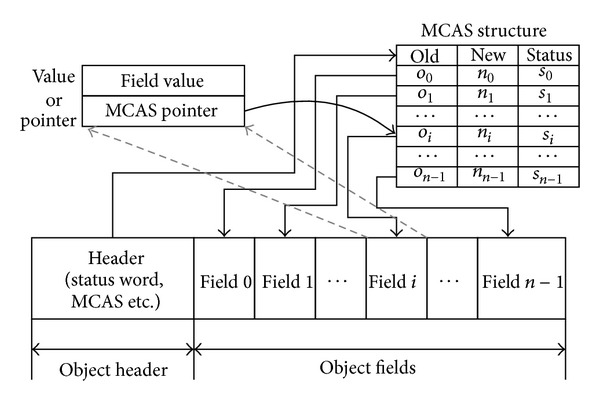
Layout of MCAS-based extended object model.

In order to meet concurrency requirement we extended the object model with MCAS structure (see Figure 3), which keeps all necessary information for each field during the copying process. Each of the structures contains three elements, an old value that is held by the corresponding field of the origin object, a new value to be copied to the target object, and a status word that indicates the copying status. And the object header is extended with a reference by which the MCAS structure can be easily accessed. Besides that there are two different meanings for the field among different copying stages. The field value will be replaced with a pointer to MCAS item when copying of the associated field is complete. All the modifications to the fields are only permitted in the MCAS structure during the concurrent copying process.

**Figure 4:**
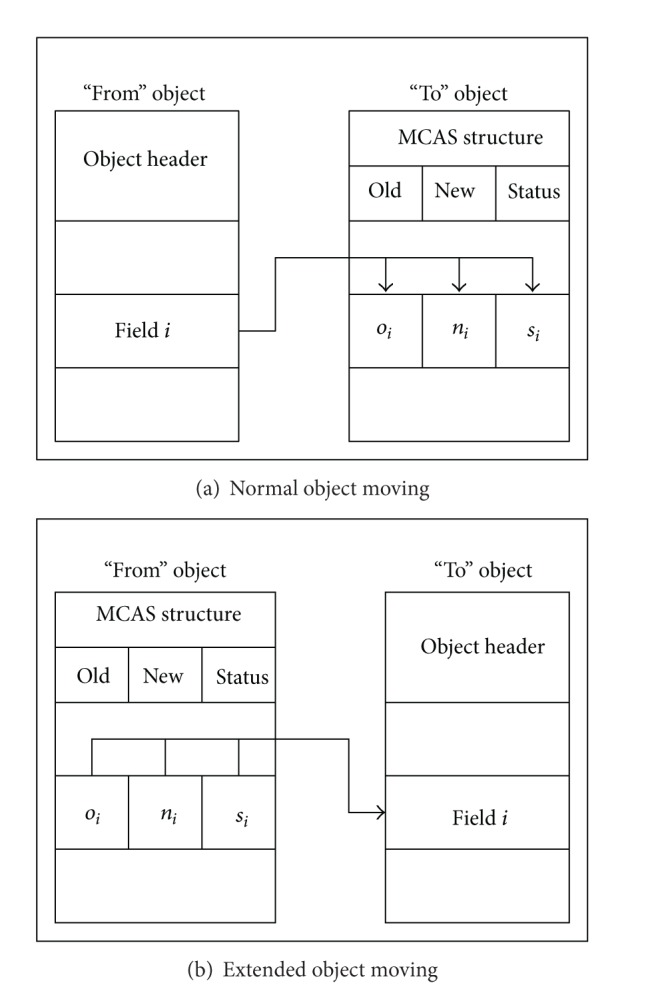
Illustration of asymmetric copying.

The MCAS-based extended object makes the synchronization easy for concurrent object copying process. Therefore, the only difficulty lies in the transformation between MCAS-based extended object and normal object. Moreover, it is unnecessary to keep both origin and replica in the MCAS-based extended form; as long as one of them is extended, the correctness of the concurrent copying process will be guaranteed. This process is called asymmetric copying as showed in Figure 4. Obviously, there exist two kinds of copying; one is copying from normal object to MCAS extended object, which is denoted as CopyNormalObject. Another is copying from MCAS extended object to normal object, which is denoted as CopyExtendedObject. The difference is the place where the MCAS information locates in. Concurrency is guaranteed in both of these two copying process, and the details are presented in Section 3.3. In order to provide a better understanding of the mechanism, four types of object mentioned in this paper are simply summarized below.

- *ExtendedObject*. The object that associates with a MCAS structure, as showed in Figure 3.
- *NormalObject*. Thin form of the extended object without MCAS structure.
- “*From” Object. *The original object that is going to be moved.
- “*To” Object*. The target copy of “from” object.

### 3.3. Details of the Copying Process

The CoypNormalObject procedure described in Procedure 1 performs the copying of normal object to a MCAS extended object. The collector first allocates an ExtendedObject with the given address in the memory. Then each status in MCAS structure is set to 0 to indicate that all of the up-to-date values of the fields are located in the “from” object. Then the copying process starts; the collector moves the fields of origin object to the target object one by one. During the copying process, the collector needs to check whether the status of the current filed in the MCAS structure is 0. If so, the collector employs a CAS operation on both field and status simultaneously. Otherwise, it skips the current field and continues copying with the next one. When all the fields are visited by the collector, the process of the copying for this object completes. For mutator, in order to achieve the up-to-date field it should check the corresponding status in the MCAS structure before reading it. Moreover, all the modifications by the mutator thread are only allowed on the extended object. Therefore, the mutator thread has to employ a CAS operation to atomically modify the field and its status.  Procedure 2 describes the barrier pseudocode for the mutator thread.

The CopyExtendedObject procedure is different from CopyNormalObject; the most essential factor is that the status words are in the “from” object.  Procedure 3 describes this procedure, which starts by initialing a target normal object in the given address of the memory. But this time the values of status in the MCAS structure should be set to 1. Compared with previous copying procedure, the collector only moves the fields in sequence from origin object to target object without status checking. During this process, collector thread employs CAS operations on extended object to ensure that the field and status are atomically updated. If the CAS operation failed, the collector should recopy the field and retry the CAS until success is reached. The modification to the object by the mutator is different from that of CopyNormalObject procedure. Mutator thread needs to check the status of the field, and the modification can only be performed when the status is 0. Otherwise, the mutator thread atomically updates the field and status by a CAS operation and keeps the value of the status remains 1. If the CAS operation fails, the mutator should retry this and choose the right copies of the object to modify by checking the current status. The barrier pseudocode for mutator thread is described in Procedure 4.

The mechanism of how to calculate the new address of the replica object during the compaction process is not described here. We only focus the concurrent copying process, and Kermany and Petrank have presented a rearrange mechanism for their proposed compressor in [6].

### 3.4. Discussion of Concurrent Handling

In this section, we discuss how the proposed mechanism maintains the consistency during the concurrent copying process. In order to facilitate analysis, we do not consider the situation with multiple collectors that execute copying task on one object. When a field of an object is being moved by the collector thread, the mutator thread may simultaneously access that field. We give the following constraints during the concurrent copying process: (1) the latest version of the field should be guaranteed to be obtained whenever the mutator or the collector accesses it. (2) Update of the field by the mutator should not be getting lost when the modification is the up-to-date one. (3) The status and its corresponding field should be simultaneously modified so as to ensure that the status word correctly indicates the latest version of its corresponding field. For the third constraints, we achieve this goal by employing CAS atomic operations on the extended objects. When the objects are in extended form, each field is attached with a status word in the MCAS-based structure. Since our implementation of MCAS supports arbitrary words synchronization, the field and its corresponding status in MCAS structure are not required to be layout continuously in memory. Status word in MCAS structure can be used to indicate where latest version of the corresponding field in kept during the copying process. Of course MCAS operations may fail if there is a change among the field and the status.

The failure of single word CAS does not affect the copying of collector in the CopyNormalObject procedure. The failure means that the target field in the extended object has been ever modified by the mutator thread which preempted the collector thread previously. Therefore, there is no need for the collector to copy the field which the collector holds which is an old version; otherwise, the update will get lost, while the mutator's strategy for dealing with the failure of CAS for extended object in the CopyNormalObject is different. The mutator thread has to guarantee the success of update operation for the mutator that holds the up-to-date field value. Therefore, the mutator retries the update operation until the CAS succeeds. However, the situation changed in the CopyExtendedObject procedure. For mutator the failure of CAS means that the target field has been copied as well as the value of status being set to 0. In this case, the update of the field is required to be migrated to “to” object by mutator.

## 4. Evaluation

Our experimental setup is summarized in Table 1. We used SPECjvm2008 and Dacapo [7] benchmark suites to evaluate the performance of our collector.

**Figure 5:**
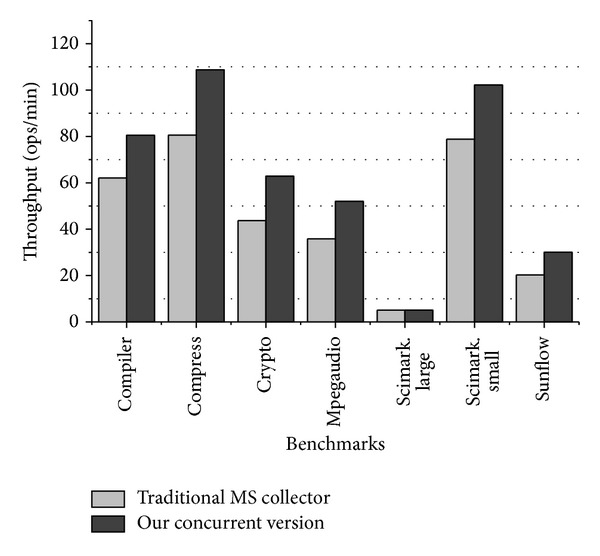
Throughput measurement results with SPECjvm2008 benchmark suite.

Throughput ability of the collector is evaluated via SPECjvm2008 benchmark suite. The SPECjvm2008 produces results in ops/min (operations per minutes) metric that reflects the rate at which the system was able to complete invocations of the workload of that benchmark. We compared our concurrent garbage collector with the traditional mark-sweep collector.  Figure 5 depicts the throughput measurement results.

**Figure 6:**
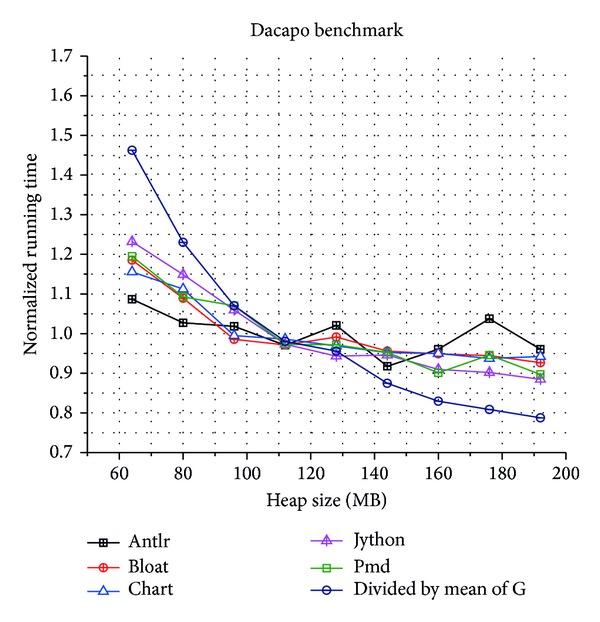
Dacapo benchmark results for our CGC with different heap sizes.

**Figure 7:**
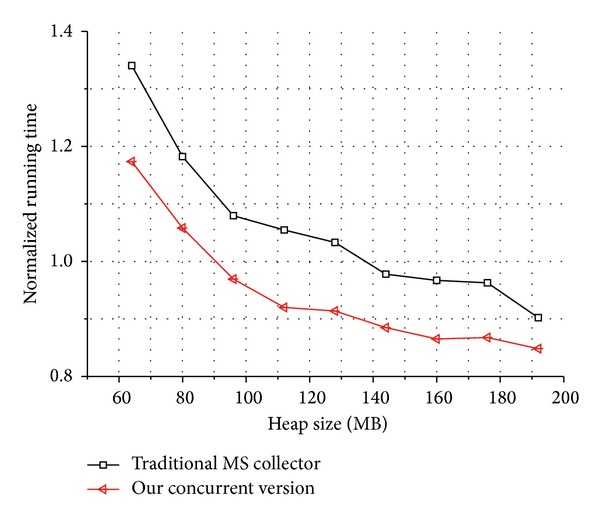
Normalized execution times for Dacapo benchmarks, with variable sized heap. Lower running time means better performance of the collector.

In order to understand the behavior of our collector, we tested it on varying heap sizes. For the Dacapo suite, we started with a 64 MB heap size and increased it by 16 MB until reaching a final size of 192 MB. The results are showed in Figure 6. Finally, Figure 7 depicts the compared results of total running time between our concurrent GC and the mark-sweep GC that with a compaction phase and lower running time means better performance.

## 5. Related Work

The first concurrent, incremental, lock-free stack scanning mechanism for garbage collector was presented by Kliot et al. [3]. The proposed mechanism allows high responsiveness and supports programs that employ fine synchronization to avoid locks. And the solution imposes a negligible overhead on the program execution. The mechanism was implemented in bartok compiler, but the extension for moving collectors was only designed without an implementation. And in [8] McGachey combined transactional memory and concurrent GC to produce a synergy that improves scalability while eliminating the annoyance of user perceivable pauses.

Pizlo et al. recently presented three different approaches for concurrent defragmenting real-time garbage collectors [9]. The STOPLESS is the first collector that provides real-time responsiveness while preserving lock-free and supporting modern parallel platform. Their CHICKEN and CLOVER collectors are both built on simple design ideas; therefore, they can obtain reduced complexity over STOPLESS. These two collectors achieve high responsiveness and low overhead although there are some design disfigurements in both of the two collectors; for example, the lock-freedom might be lost with a small probability in CLOVER.

Recently, several approaches for real-time garbage collectors using special designed hardware have been presented. A soft real-time collector for Azul systems was proposed by Click et al. [10]. They use special hardware to support access migration during the object copying process. And Schoeberl and Puffitsch [11] also presented a nonblocking real-time GC which was implemented in hardware.

Works on real-time garbage collector began with Baker [12], and his approach has been the basis for most of the later work. Cheng [13] introduces the notion of minimum mutator utilization and built a multiprocessor collector with high observed MMU. Bacon et al. [14] provide basic principles of the Metronome collection algorithm; they have made the collector to meet real-time bounds by limiting copying and incrementalizing defragmentation, and the extension of the Metronome is presented in [15].

## 6. Conclusions

We have studied how to design a lock-free concurrent garbage collector on multicore platform. Our goal is to improve the efficiency of garbage collection algorithm and to make full use of the resources of multicore platform. In this paper we have mainly studied two issues of designing concurrent garbage collectors, stack scanning and heap compaction. In order to overcome the drawbacks of the lock-based algorithms, we have introduced a lock-free solution for implementing highly concurrent garbage collectors. We have presented a lock-free methodology for constructing concurrent stack scanning and concurrent heap compaction via CAS and multiword CAS primitives. The experimental results show that our concurrent strategy achieves performance comparable with the traditional designs.

## Figures and Tables

**Algorithm 1:**
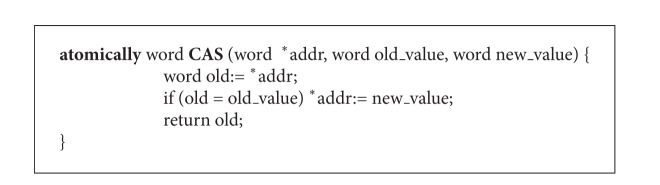
Abstract definition of CAS primitive.

**Algorithm 2:**
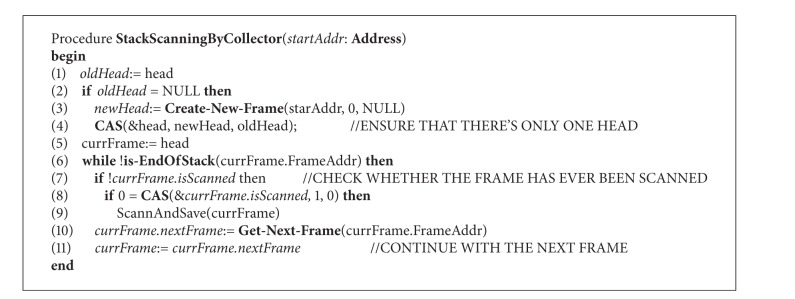
Stack scanning procedure of collector thread.

**Algorithm 3:**
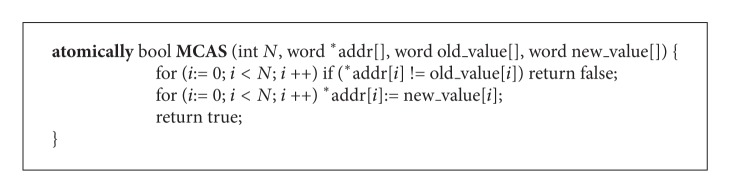
Abstract definition of multiword CAS primitive.

**Procedure 1:**
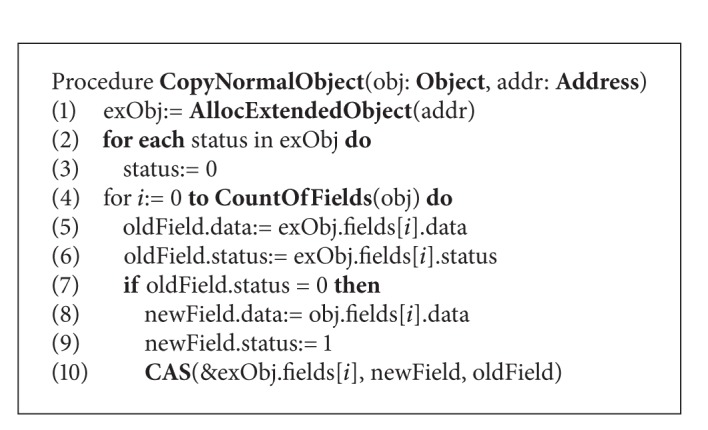
CopyNormalObject procedure (procedure code for collector).

**Procedure 2:**
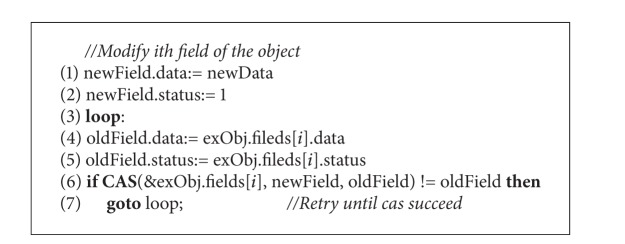
CopyNormalObject procedure (barrier code for mutator).

**Procedure 3:**
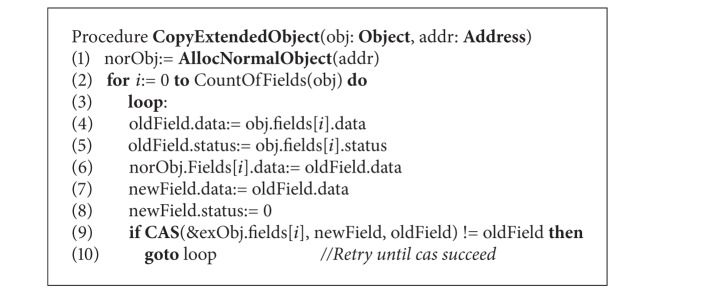
CopyExtendedObject procedure (procedure code for collector).

**Procedure 4:**
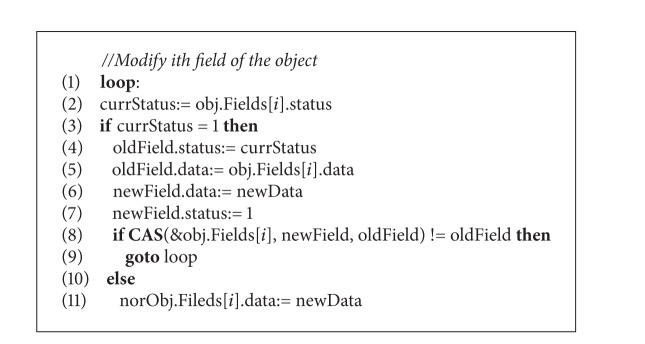
CopyExtendedObject procedure (barrier code for mutator).

**Table 1:** Measurements setup details.

Items	Descriptions
Platform	Intel Core2 Q8300 CPU, 4 cores, 2.50 GHz, 2 GB physical memory Ubuntu 10.04 Linux kernel 2.6.32-29-generic

Benchmarks	Dacapo (antlr bloat chart jython pmd xalan) SPECjvm2008 (compiler compress crypto mpegaudio scimark sunflow)

JVM	Jikes Research VM 3.1.1
